# Ozonated Oil for Rheumatic Diseases

**DOI:** 10.34172/apb.025.44040

**Published:** 2025-10-15

**Authors:** Jozélio Freire de Carvalho, Ana Tereza Amoedo Martinez

**Affiliations:** ^1^Núcleo de Pesquisa em Doenças Crônicas não Transmissíveis (NUPEC), School of Nutrition from the Federal University of Bahia, Salvador, Brazil; ^2^Universidade Estadual de Feira de Santana, Feira de Santana, Brazil; ^3^Novaclin, Grupo CITA, Salvador, Brazil

## To Editor,

 Ozone therapy has emerged as a highly effective antimicrobial approach, widely used in clinical practice due to its well-documented therapeutic benefits.^1^ Its primary mechanism of action involves potent oxidizing properties capable of disrupting bacterial cell walls and cytoplasmic membranes, combined with significant anti-inflammatory effects.^1^ Ozone therapy has also been extensively applied in the management of inflammatory conditions, particularly osteoarthritis (OA). This treatment appears to exert anti-inflammatory and analgesic effects, potentially through the modulation of oxidative stress and the improvement of tissue oxygenation, which together contribute to reduced discomfort and enhanced mobility.^2^ A recent meta-analysis of eight randomized controlled trials involving 718 participants demonstrated a significant short-term reduction in knee pain within 3 to 6 months following ozone administration.^2^

 Ozone can be delivered via multiple routes, including intra-articular, intramuscular, subcutaneous, rectal, and topical applications using ozonated oil (OzO) (Table 1). To explore the potential of a non-invasive ozone delivery method, we reviewed the literature regarding the use of OzO in rheumatic diseases.

**Table 1:** Summary of the main delivery routes used in ozone therapy.

**Delivery routes**	**Clinical use examples**	**Evidence in osteoarthritis / rheumatic diseases**	**Advantages**	**Limitations / Risks**
Intra-articular	Direct injections into knee or hip	Meta-analyses: significant pain reduction (3–6 months)	Rapid local action; strong anti-inflammatory effect	Invasive procedure; risk of pain or infection
Intramuscular / Subcutaneous	Used occasionally for chronic pain	Limited evidence	Easy to apply	Variable absorption; uncertain systemic effect
Rectal (insufflation)	Applied in inflammatory or systemic conditions	Rarely studied in OA	Well tolerated, systemic effect	Requires equipment; limited patient acceptance
Topical (ozonated oil)	Applied over painful joint, twice daily	1 RCT (n = 80): significant pain reduction only in severe OA (p = 0.021)	Non-invasive, safe, home-based use	Limited evidence; lack of standardized ozone dosage
Ozonated water / compresses	Wounds, ulcers, skin inflammation	No OA data	Low cost, safe	Low stability; mainly restricted to skin/mucosa

 A comprehensive search of PubMed, SciELO, and LILACS databases was conducted, covering publications from 1965 to May 2024, with no language restrictions. Studies were excluded if they were reviews or focused on in vivo or in vitro experiments. After screening titles and abstracts, only one study met the inclusion criteria.

 The selected study, conducted by Anzoli et al,^3^ was a randomized, triple-blind, placebo-controlled trial involving 80 patients with OA (76% female; mean age 64.57 ± 8.83 years). This study evaluated the effects of twice-daily topical application of OzO in 37 OA patients compared with a placebo group of 43 patients over two months. Pain relief was assessed using the Western Ontario and McMaster Universities Osteoarthritis Index (WOMAC) and the Visual Analog Scale (VAS). While both groups experienced significant pain reduction, improvements in severe OA were observed exclusively in the OzO-treated group.^3^ After 60 days, the OzO group improved to about 60 points compared to 68 points in the placebo group, with a significant reduction observed only in severe OA (*P* = 0.021 vs. *P* = 0.345, respectively), while mean CRP levels in the OzO group decreased from 14.62 ± 5.75 mg/L to 12.81 ± 5.5 mg/L and in the placebo group from 6.63 ± 2.71 mg/L to 4.37 ± 1.31 mg/L.^3^ However, the study did not perform quantitative assessments of ozone content in the OzO or in biological samples, precluding determination of the actual ozone concentration associated with the intervention. In clinical practice, OzO is typically applied by gentle massage over the affected joint, typically twice daily for several weeks, and is generally well tolerated.

 Regarding mechanisms of action, the anti-inflammatory effects of OzO appear to result from multiple molecular mechanisms. The ozonides and peroxides generated during the ozonation process induce a mild oxidative stress that activates the Nrf2 pathway, leading to the transcription of endogenous antioxidant enzymes (such as SOD, CAT, GPx, and HO-1), while simultaneously inhibiting the pro-inflammatory NF-κB pathway and reducing the expression of cytokines and COX-2.^4^ Experimental data also indicate that topical OzO reduces key inflammatory mediators, including IL-6, IL-1β, TNF-α, and IFN-γ. Furthermore, OzO has been shown to enhance tissue repair and wound healing by modulating reactive oxygen species (ROS), promoting local growth factor release (e.g., TGF-β, PDGF, VEGF), and improving oxygen supply to the affected tissue.^4^ Collectively, these pharmacodynamic mechanisms support the rationale for its topical use in inflammatory and degenerative joint conditions.

 In conclusion, only one study to date has investigated the effects of OzO in rheumatic diseases, specifically O, reporting improvements in both subjective and objective measures. Based on these findings, future research on OzO should focus on quantifying its ozone-derived compounds, establishing standardized dosages and treatment durations, and conducting long-term, large-scale clinical trials to confirm its efficacy, safety, and underlying the mechanisms of action in OA.

## Competing Interests

 Authors declare that they have no conflicts of interest.

## Data Availability Statement

 All data of our study is available at request.

## Ethical Approval

 The present article performed a literature search and did not need any Ethical approval.
